# Early Substance Use Cessation Improves Cognition—10 Years Outcome in First-Episode Psychosis Patients

**DOI:** 10.3389/fpsyt.2019.00495

**Published:** 2019-07-12

**Authors:** Melissa A. Weibell, Jan Olav Johannessen, Bjørn Auestad, Jørgen Bramness, Kolbjørn Brønnick, Ulrik Haahr, Inge Joa, Tor Ketil Larsen, Ingrid Melle, Stein Opjordsmoen, Bjørn Rishovd Rund, Jan Ivar Røssberg, Erik Simonsen, Per Vaglum, Helen Stain, Svein Friis, Wenche ten Velden Hegelstad

**Affiliations:** ^1^TIPS Centre for Clinical Research in Psychosis, Division of Psychiatry, Stavanger University Hospital, Stavanger, Norway; ^2^Faculty of Health, University of Stavanger, Stavanger, Norway; ^3^Department of Mathematics and Physics, University of Stavanger, Stavanger, Norway; ^4^Research Department, Stavanger University Hospital, Stavanger, Norway; ^5^Norwegian National Advisory Unit on Concurrent Substance Abuse and Mental Health Disorder, Innland Hospital Trust, Hamar, Norway; ^6^Institute of Clinical Medicine, Faculty of Medicine, University of Oslo, Oslo, Norway; ^7^Institute of Psychiatry, University of Bergen, Bergen, Norway; ^8^Division of Mental Health and Addiction, Oslo University Hospital, Oslo, Norway; ^9^Department of Psychology, University of Oslo, Oslo, Norway; ^10^Vestre Viken Hospital Trust, Drammen, Norway; ^11^Psychiatric Research Unit, Psychiatry Region Zealand, Slagelse, Denmark; ^12^Institute of Clinical Medicine, Faculty of Health and Medical Sciences, University of Copenhagen, Copenhagen, Denmark; ^13^Department of Behavioural Sciences in Medicine, University of Oslo, Oslo, Norway; ^14^School of Social and Health Sciences, Leeds Trinity University, Leeds, United Kingdom

**Keywords:** psychosis, substance use, cognition, neurocognition, first-episode psychosis

## Abstract

**Background:** Cognitive impairment may be a risk factor for, as well as a consequence of, psychosis. Non-remitting symptoms, premorbid functioning, level of education, and socioeconomic background are known correlates. A possible confounder of these associations is substance use, which is common among patients with psychosis and linked to worse clinical outcomes. Studies however show mixed results for the effect of substance use on cognitive outcomes. In this study, the long-term associations of substance use with cognition in a representative sample of first-episode psychosis patients were examined.

**Methods:** The sample consisted of 195 patients. They were assessed for symptom levels, function, and neurocognition at 1, 2, 5, and 10 years after first treatment. Test scores were grouped into factor analysis-based indices: motor speed, verbal learning, visuomotor processing, verbal fluency, and executive functioning. A standardized composite score of all tests was also used. Patients were divided into four groups based on substance-use patterns during the first 2 years of treatment: persistent users, episodic users, stop-users, and nonusers. Data were analyzed using linear mixed effects modeling.

**Results:** Gender, premorbid academic functioning, and previous education were the strongest predictors of cognitive trajectories. However, on motor speed and verbal learning indices, patients who stopped using substances within the first 2 years of follow-up improved over time, whereas the other groups did not. For verbal fluency, the longitudinal course was parallel for all four groups, while patients who stopped using substances demonstrated superior performances compared with nonusers. Persistent users demonstrated impaired visuomotor processing speed compared with nonusers. Within the stop- and episodic use groups, patients with narrow schizophrenia diagnoses performed worse compared with patients with other diagnoses on verbal learning and on the overall composite neurocognitive index.

**Discussion:** This study is one of very few long-term studies on cognitive impairments in first-episode psychosis focusing explicitly on substance use. Early cessation of substance use was associated with less cognitive impairment and some improvement over time on some cognitive measures, indicating a milder illness course and superior cognitive reserves to draw from in recovering from psychosis.

## Introduction

Cognitive impairment is a core feature of schizophrenia. It is observed in the majority of patients (1, 2), often present before the onset of psychosis and is also prevalent in non-affected relatives (3, 4). It is associated with negative symptoms such as apathy and flat affect (5, 6), and several studies have shown an association with poorer clinical and functional outcomes (7). Previous studies report deficits in both processing speed and episodic memory (8), as well as working memory, executive functions (9), and attention (10). One meta-analysis (11) showed moderate to large effect sizes across all cognitive domains, with impairments being more pronounced in older and more chronic patients. Correlates of cognitive impairments include premorbid intellectual functioning, level of education, social functioning, and socioeconomic status (12–14). It has also been suggested that the prevalent long-term use of antipsychotic medication in schizophrenia spectrum disorders could compromise cognitive functioning (15, 16). However, studies with short follow-up intervals have also found indications of cognitive improvement associated with the use of antipsychotics (17–19).

A possible confounder in the relationship between cognitive impairments and outcome is substance use, which is common in patients with psychosis. Reported prevalence rates of concurrent substance use converge on 50%, significantly higher than rates in the general population (20–22). Experimental studies have shown that tetrahydrocannabinol transiently induces psychotic symptoms in a dose-dependent manner and cognitive impairment in healthy individuals (23). Cannabis use is also consistently associated with more cognitive impairments in studies of schizophrenia (24); however, there are some contradictory findings. Several cross-sectional studies have found superior performance in visual memory, working memory, and executive functioning (25–30), attention (31), and, in overall, cognitive task performance in substance-using compared with the performance in non-using patients (12, 13, 32). Long-term longitudinal studies of cognition in psychosis are scarce, and very few extend beyond a 5-year follow-up (33–42). Overall findings indicate stable impairment over time. Studies focusing explicitly on the role of substance use appear to be lacking.

Several studies have reported that continued substance use leads to poorer outcomes than those who stop substances early on in their course of treatment (43, 44). Cessation of use is associated with improvements in symptoms, depression, and functioning (45–47). To our knowledge, no studies have focused on substance-use cessation and the effect on cognition in first-episode psychosis (FEP) patients.

The early Treatment and Intervention in Psychosis (TIPS) study is a prospective, longitudinal study that originally sets out to investigate the relationship between duration of untreated psychosis (DUP) and outcome in FEP patients. It includes a very rich database of the development of significant clinical characteristics from the first week of treatment. We have previously shown that substance users who stopped using during the first 2 years of treatment show a different illness trajectory than those who continue using or stopped using at a later point in time (47). Substance users had better social premorbid functioning than nonusers (NUs) (48). Cognition, in general, appeared to be stable over the first 10 years in treatment (39) in our sample, also with regard to clinical subsamples (39) and using improved statistical methods (49). Improved verbal memory and learning at 1- and 2-year follow-up was associated with fewer relapses during the first year of treatment (50), and follow-up analyses of subsamples suggested that patients who relapsed during the first year of treatment had different cognitive trajectories over the 10-year period (39).

The aim of the current study is to examine the long-term (10-year) associations between substance use and cognition as well as the effect of early substance-use discontinuation in the TIPS sample. Based on our extensive data material, we will also take into account potential predictive or confounding factors such as premorbid functioning, clinical symptoms, and diagnostic groups (narrow versus broad schizophrenia spectrum).

## Material and Methods

### Study Design

The TIPS study is a prospective, longitudinal follow-up of a large, clinical epidemiological cohort recruited consecutively over 4 years from four Scandinavian health care sectors during 1997–2000. These include two sectors in Rogaland County, Norway, the Ullevål sector in Oslo County, Norway, and a sector from Roskilde County, Denmark. The combined estimated population at the start of the study period was 665,000 inhabitants. Health care services were catchment area based and publicly funded in all sectors. The areas were similar sociodemographically (e.g. urbanicity, mean educational and income levels, and opportunities for employment) (51). Patients from all areas were treated according to a 2-year standard treatment protocol that included antipsychotic medication, supportive psychotherapy, and multi-family psycho-education.

### Participants

The sample consisted of FEP patients with *Diagnostic and Statistical Manual of Mental Disorders*, 4th Edition schizophrenia, schizophreniform disorder or schizoaffective disorder (“narrow schizophrenia spectrum”), delusional disorder, mood disorder with mood-incongruent psychotic features, brief psychotic disorder, or psychosis not otherwise specified (“broad schizophrenia spectrum”) (51, 52). Participants had to reside in one of the participating sites and were 15–65 years of age in Rogaland or 18–65 years in Oslo/Roskilde and within the normal range of intellectual capacity (Wechsler Adult Intelligence Scale—Revised-based IQ estimate >70). Participants were included between 1997 and 2001 (baseline) and followed up at 1-, 2-, 5-, and 10 years. Twenty-three percent of those who were eligible declined participation. Within the group of 301 who consented to participate, the current sample consists of those who completed cognitive testing at baseline (*n* = 218) who had data for substance-use grouping (*n* = 195). There were no statistically significant differences in symptom levels, age, gender, premorbid functioning, or diagnostic distribution between those who did and those who did not complete testing at baseline. A total of 87% completed at least two neuropsychological tests, and 22% completed all five follow-ups. There were 138, 137, 82, and 85 participants who completed neurocognitive testing at each follow-up point. Dropout analyses did not show any statistical differences with regard to diagnoses, gender, duration of untreated psychosis (DUP), substance use, symptom levels, premorbid functioning, or age at 1-, 2-, 5-, or 10-year follow-up. However, 5- and 10-year follow-up dropouts had higher excitative symptom component scores at baseline. Also, participants who dropped out in the course of the study had better scores on the trail making tests (visuomotor processing) compared with those who only completed one test (*t* = 3.7; df: 44.4; *p* < .001).

### Assessments

The Structured Clinical Interview for the *Diagnostic and Statistical Manual of Mental Disorders*, 4th Edition (severe combined immunodeficiency) (53) was used for diagnostic purposes. All included patients were assessed using the global assessment of functioning (GAF) split into symptom and function scores (54). Demographic data, including family history of mental illness, was collected for all study-eligible patients. DUP was measured as weeks from the emergence of positive psychotic symptoms to the start of adequate treatment, defined as structured treatment with antipsychotic medication or the admission to psychiatric wards for psychosis. A few non-admitted patients started outpatient psychotherapy structured and directed toward psychosis but did not want medication initially. For these patients, start of psychotherapy was regarded as the start of adequate treatment. Symptom levels were measured by the positive and negative syndrome scale (PANSS) (53), scored on five symptom domains: positive, negative, cognitive, depressive, and excitative symptoms (55). Items constituting these components are as follows: positive component items P1 delusions, P3 hallucinatory behavior, P5 grandiosity, P6 suspiciousness, and general scale item G9 unusual thought content; negative component N1 blunted affect; N2 emotional withdrawal; N3 poor rapport; N4 passive withdrawal; and general scale items G7 motor retardation, G13 disturbance of volition, and G16 active social withdrawal; cognitive component items P2 conceptual disorganization, N5 difficulty in abstract thinking, N6 lack of spontaneity and flow of conversation and general scale items G10 disorientation, G11 poor attention and G15 preoccupation; depressive component general scale items G1 somatic concern, G2 anxiety, G3 guilt feelings, G4 tension and G6 depression; and excitative component items P4 excitement, P7 hostility, and general scale items G8 uncooperativeness and G14 poor impulse control. Onset of FEP positive symptoms was defined as a PANSS score of 4 or higher on any of the PANSS positive component items; not previously receiving adequate treatment for psychosis defined as antipsychotic medication of 3.5 haloperidol equivalents for 12 weeks or until remission of psychotic symptoms. Remission was defined as subthreshold symptoms for at least 7 days, whereas relapse involved reappearance of positive symptoms (items 1, 3, 5, 6, or general scale item 9) for at least 7 days. Stable remission was defined as no relapse in the first year after admission (53–55).

Premorbid functioning was measured by the premorbid adjustment scale (56), covering two areas of functioning—school adaptation and socialization—described through initial childhood level and subsequent change (57). Scores ranged from 1 through 6 with higher scores indicating more impairment. A premorbid adjustment scale change score was calculated as the difference between childhood scores and the last score available, to indicate decline or improvement over time (56, 57).

Length of treatment was split into number of weeks of antipsychotic medication and the number of weeks of psychosocial treatments measured as the sum of weeks with uninterrupted psychosocial treatments with a frequency of once every fortnight or more for the first 5 years or once a month between 5 and 10 years.

### Neurocognitive Measures

Neurocognitive tests were administered by clinical psychologists trained in standardized assessments or by research assistants supervised by a senior psychologist.

The five domains of neurocognitive functioning were:

*Verbal Learning and Delayed Recall (VL/VL index):* The California verbal learning test (CVLT) was used to assess this domain, and the revised version of CVLT was used at 10-year follow-up (58). The number of words and trials were identical to the original version used at previous assessments, while scores were obtained for total immediate recall (the mean sum of trials 1–5), errors (the mean sum of trials 1–5), delayed free recall, and perseverative responses. Combining raw scores obtained from these two test versions in the same analysis was justified as equivalency in total learning, and long-delay free recall raw scores is reported in healthy individuals (58).
*Motor Speed (MS/MS index):* The finger tapping test with both hands was used, and the mean score for both the dominant and nondominant hand was calculated.
*Visuomotor Processing [trail making (TM) index]:* Trail making (A and B) was used, with the scores representing total time for completion of both parts A and B.
*Executive Function index:* Executive Function index was assessed by the Wisconsin card sorting test, PC version (59). The scores were “categories completed,” “perseveration,” “trials to first category,” and “failure to maintain sets.”
*Verbal Fluency* index was assessed by the controlled oral word association task (60), where the sum mean scores for F-words, A-words, and S-words were used. At baseline, this domain also included measures from the digit span (with distractor) and continuous performance tests (number of hits) (61), but these were not repeated at 10-year follow-up.

For all tests, a *z* score was calculated based on mean scores at baseline. Except for finger tapping, indices were moderately correlated. The four indices (CVLT, TM, Wisconsin card sorting test, and controlled oral word association task) were therefore added together and averaged to form a composite index.

All cognitive ratings were done blind to the substance-use group affiliation of the participants. Reliability of GAF, DUP, and diagnosis was found satisfactory throughout the study. The results of the reliability assessments have been reported previously (62, 63).

### Measurement and Classification of Substance Use

Substance and alcohol use was measured by the alcohol and drug use scale (64) using a scale from 1 to 5 (1 = no use; 2 = use without impairment; 3 = abuse; 4 = dependence; 5 = dependence with institutionalization). All commonly used illegal psychoactive substances were included in the assessment. We did not include tobacco, caffeine, or alcohol in our definition of substance use, as these follow different treatment paths and sequelae.

Patients were dichotomized into users or not users, where “use” was defined as any score >1. Abstinence is a culturally relevant concept in Norway, where substance use is largely restricted to subgroups, with any use being considered harmful. Patients were assessed concerning pattern of substance use at all follow-up points. At 5-year follow-up, we also did a retrospective assessment of substance use at 3 and 4 years based on patient information and medical charts. Patients’ substance use changed most during the first 2 years after inclusion; thus, this interval was chosen for grouping. This interval is consistent with prior studies (65–68).

For analyses, we grouped patients into a) nonusers (NUs), i.e. patients who had never used, b) stop-users (SUs), c) episodic users (EUs), and d) persistent users (PUs). Patients who had only “no-use” measurements during the first 2 years of follow-up were defined as nonusers (NUs). Patients who had used at baseline and then not use for at least two consecutive measurements, i.e. at 1 and 2 years of follow-up, were defined as stop-users (SUs). Persistent users (PUs) used at all follow-up points, and episodic users (EUs) had various other substance-use patterns. This four-group solution was chosen based on recent studies that have shown that around half of substance-using patients who stop using appear to have less severe symptoms than those who continue (45). Merging previous substance users with NUs does not aid in understanding the impact of ceasing substance use on patient trajectories or prognosis.

### Statistics

Statistical analyses were carried out using SPSS version 22.0 (69) and R version 3.4.3 (70).

Differences between groups at baseline were described using frequencies and percentages for categorical variables and means and standard deviations or medians and ranges for continuous variables. Comparisons between groups were made using chi-squared tests for categorical data and student t-tests for independent samples for continuous data. All tests were two-tailed.

To investigate the effect of substance abuse on performance over time, linear mixed effects models were used. The model uses maximum likelihood estimation to manage dropout to a certain degree. This is based on the assumption of dropout at random, that is, the probability of dropout is independent of future but may be dependent on previous history, which may be reasonable in this situation. Separate models were estimated, each with one of the cognitive index scores as the dependent variable and substance-use group as categorical predictor. Covariates were based on baseline differences: age, gender, years of education, and premorbid academic adjustment (**Table 1**). Furthermore, based on the literature, diagnostic category (narrow schizophrenia spectrum disorder or not) and DUP (log transformed due to skewed distribution) were included. Interaction between time and group was included in order to investigate whether change in neuropsychological test scores developed differently in the different groups. Furthermore, the interaction between narrow schizophrenia spectrum diagnoses and group was examined to determine whether narrow schizophrenia diagnoses are associated with different effects on the neuropsychological tests in the substance-use groups. The large data set justifies the number of parameters in the models. Random intercept and AR (1) was used to achieve a satisfactory model for correlation between longitudinal measurements within individuals. The executive function and TM indices were severely skewed to the left. In order to achieve a robust analysis, these data were log transformed after inverting the scale and adding a constant to assure positive values only.

**Table 1:** Baseline characteristics at study inclusion and at 10 years of first-episode psychosis patients across patterns of substance abuse.

*N* = 195	No (NU) *N* = 106		Stop (SU) *N* = 26		Episodic (EU) *N* = 33		Persistent (PU) *N* = 30		Analysis	
	N	%	N	%	N	%	N	%	Chi^2^	df
Male*	48	45	20	77	20	61	23	77	15.1	3
**Diagnosis at inclusion**										
Schizophrenia spectrum	71	67	18	69	26	79	21	70		6
Affective	20	19	2	8	3	9	3	10		6
Other”	15	14	6	23	4	12	6	20		
	Median	Range	Median	Range	Median	Range	Median	Range	F	Df***
DUP (weeks)**	8	0–520	9	0–416	17	0–468	16	1–555	1.6	191
	Mean	SD	Mean	SD	Mean	SD	Mean	SD	F	Df***
Age****	30.8	10.3	27.5	8.3	21.9	4.0	22.5	4.3	13.2	191
Premorbid adjustment, last score										
Social	1.8	1.5	1.5	1.2	1.9	1.3	1.9	1.6	0.4	191
Academic*****	2.2	1.4	2.3	1.3	2.5	1.2	3.0	1.4	2.8	191
**PANSS and GAF baseline**										
Positive	15.3	4.4	16.1	4.9	14.7	3.6	15.2	4.7	0.4	190
Negative	20.2	9.8	20.4	8.1	22.3	9.2	21.3	7.5	0.4	189
GAF function.007 (SU/PU.01)	31.6	10.4	27.8	9.2	33.3	10.5	8.6	1.6	4.2	189
**PANSS and GAF at 10 years**										
Positive comp (NU/PU.035), (SU/PU.018)	8.5	4.1	7.2	3.3	10.2	4.5	11.6	5.5	4.6	130
Negative	15.9	7.2	14.6	6.3	17.9	6.9	19.5	9.6	1.9	130
GAF function 001 (SU/EU.002) SU/PU.036)	52.2	14.3	62.7	12.0	44.3	14.0	49.0	17.2	5.5	130

*p < .002; “Other diagnoses: delusional disorder (n = 7), brief psychotic disorder (n = 3), organic psychosis (n = 1), psychosis NOS (n = 10); **Reported values are median values, while analysis of variance was done with log transformed DUP values; ***All between-group degrees of freedom (df) = 3. Df reported in table concerns within-group df; ****Post hoc comparisons Scheffe test, pairwise comparisons NU&EU, NU&PU p < .001; *****p < .05; ^#^Post hoc comparisons Scheffe test.

## Results

**Table 1** outlines the demographic and clinical characteristics of the sample. Substance users (all) at baseline were more likely to be male than NUs, had poorer premorbid academic functioning, and shorter length of education. NUs were significantly older at presentation than EUs, and PUs (*p* = 0.001 in both groups), but not SUs. No differences in terms of diagnostic distribution or DUP were found between groups. There were no differences in positive or negative symptoms on the PANSS or in GAF function scores between groups. Not outlined in the table; there were no group differences for family history.

The positive PANSS component scores differed among groups at all follow-up points post-baseline, with PUs exhibiting significantly higher symptom levels. The duration of use of antipsychotics or psychotherapy over the 10-year period did not differ between users and NUs. There were differences in time spent in hospital, both on a yearly basis (*p* < 0.048) and cumulatively (*p* = 0.048). In addition, substance users spent more time in psychosis, both per year (CU > NU *p* = 0.011; PU > SU *p* = 0.024) and cumulatively (CU > NU *p* = 0.011; PU > SU *p* = 0.024). Mean values and 95% CI for the neurocognitive indices shown over time in the groups are provided in the **Supplementary Material**.

### Motor Speed

There were no group differences at baseline for motor speed (**Table 2**). LME modeling showed that development over time was significantly different between groups. SUs performed better over time (*t* = 2.20; df 433; *p* = 0.03) compared with all groups. Females had lower scores over time and at baseline (*t* = −7.07; df 208; *p* = 0.001).

**Table 2:** Standardized neuropsychological test scores at baseline in first-episode psychosis patients across patterns of substance abuse.

*N* = 195	No (NU) *N* = 106		Stop (SU) *N* = 26		Episodic (EU) *N* = 33		Persistent (PU) *N* = 30		Analysis		
	Mean	SD	Mean	SD	Mean	SD	Mean	SD	F	p	Df within
Neuropsychological index scores											
Verbal learning	.17	1.1	.01	.82	-.08	.94	−.25	.95	1.4	.241	184
Verbal fluency	.03	.96	−.06	1.1	.16	.80	.14	.97	.37	.773	187
Executive function	−.43	.89	.12	.87	.06	.74	−.04	1.0	.29	.831	184
Motor speed	−.13	.96	.10	1.1	.14	1.1	.32	.89	1.9	.132	188
Trail Making	−.07	1.2	−.13	.79	−.12	.98	.42	.67	2.0	.113	183
Composite score	.01	.64	−.05	.69	.01	.49	.07	.61	.19	.906	191

### Executive Function

There were no significant group differences for executive functioning.

### Verbal Learning

All four groups perform poorer with time on verbal learning. SUs had higher scores across all follow-up points (*t* = 2.00; df 211; p = 0.05). Poorer premorbid function was associated with lower scores (*t* = −2.57; df 211; *p* = 0.01). Women performed significantly better than men at all follow-up points (*t* = 4.95; df 211; p = 0.001). Patients with narrow schizophrenia diagnoses in the SUs (*t* = −2.03; df 211; p = 0.04) and EUs (*t* = −2.76; df 211; *p* = 0.006) performed poorer than NUs.

### Visuomotor Processing

The PUs scored significantly poorer than NUs across all time points on visuomotor processing (*t* = −2.37; df 207; *p* = 0.020). Performance levels were predicted by education where shorter length (*t* = −3.58; df 207; *p* = 0.001) and poorer premorbid adjustment predicted lower scores. Higher age was also a predictor of poorer scores (*t* = 4.63; df 207; *p* = 0.001).

### Verbal Fluency

The SUs scored significantly higher than the NUs on verbal fluency, although change over time was parallel (*t* = 2.21; df 210; *p* = 0.03). There was a significant improvement over time in all groups. Longer education (*t* = 2.23; df 210; p = 0.03), better premorbid functioning (*t* = −2.75; df 210; *p* = 0.006), and female gender (*t* = 2.68; df 210; *p* = 0.008) were associated with better scores.

### Composite Score

There was no significant change over time and no significant group differences in overall performances. The composite score was significantly associated with longer education (*t* = 2.73; df 213; *p* = 0.006), better premorbid functioning (*t* = −3.98; df 213; *p* = 0.001), female gender (*t* = 2.29; df 213; *p* = 0.022), and lower age (*t* = −2.24; df 213; *p* = 0.026). Within the SUs (*t* = −2.32; df 213; *p* = 0.022) and EUs (*t* = −2.34; df 213; *p* = 0.021), the narrow schizophrenia group performed poorer.

In summary, patients who stopped using substances had higher motor speed, better verbal learning, and better verbal fluency. Persistent users performed significantly worse on visuomotor processing, while participants who had never used substances had significantly better visuomotor processing and poorer verbal fluency. For EUs and SUs, patients with narrow schizophrenia diagnoses performed significantly poorer overall.

## Discussion

This study is one of the firsts to focus on cognition, substance use, and substance-use discontinuation in a sample of FEP patients. Our study is longitudinal and includes a large and representative sample. The main finding was that those who stop using substances early have superior cognitive functioning on several measures compared with those who continue using, either persistently or episodically. Those who stop using within the first 2 years of receiving treatment do as well as, or better than, NUs.

Better performance on cognitive functioning indices were associated with better premorbid academic functioning and more years of education as well as female gender. Persistent and EUs had poorer premorbid academic functioning and were more likely to be male. However, both male gender and poor premorbid adjustment represent poor prognostic factors in psychosis. Thus, it may be challenging to disentangle the effect of poor premorbid adjustment from substance use.

For instance, the trail making test and verbal fluency both have a strong component of mental control. Trail making part B relies heavily on set-shifting ability, and verbal fluency, whereas the F-A-S measure of verbal fluency relies on efficient search skills and, hence, also mental control. Both these tests were associated with premorbid educational attainment, academic adjustment, and substance use. Furthermore, mental control is an ability that is often compromised in patients with more severe psychotic illnesses. Improvement and superior performances in those who stop using substances and worse performances in those who continue to use may therefore contribute to a growing evidence base suggesting a milder illness process in SUs. It has indeed been suggested from other studies that substance users may have better cognitive functioning than NUs and follow a different path to illness, with a separate starting point and trajectory toward psychosis. The finding that verbal fluency was impaired in those who never used substances aligns well with this: verbal fluency has repeatedly been shown to be a robust and central impairment in schizophrenia and other psychoses. Having developed psychosis in the absence of the risk factor substance use may thus be indicative of a more severe or even more endogenous illness process.

Previous findings from this and other studies (45, 66, 71) show that patients who stop using have better clinical and functional outcomes than both EUs and PUs. One may speculate that these patients lack some vulnerabilities present in other groups and that perhaps psychosis may even have been avoided in the absence of substance use.

Susceptibility to psychosis is considered familial to a certain degree, and some family studies have found deficits in verbal learning and motor speed (72, 73) in unaffected relatives. We did not find any significant difference in the rate of positive family history of mental illness in first-degree relatives between groups or diagnostic categories. In summary, our findings appear to underscore the importance of substance use as an independent risk factor and, more malleable than familial risk, trauma, and other known factors. The possibility of substantial harm reduction with early discontinuation is an important message to clinicians and provides hope for patients who struggle with addiction and psychosis.

A longitudinal study such as ours holds several methodological limitations. Retest effects in cognitive testing are one of these. However, the spacing over a 10-year period with long intervals between testing reduces training effects. Since CVLT is the most likely candidate for training effects, we also used a parallel version at the 10-year follow-up.

The rate of dropout is high, although we have compensated for this by using linear mixed model analyses that account for missing data by calculating estimates.

Our main limitation concerns the lack of means for controlling patient’s claims of substance-use cessation. This information could have provided valuable information in terms of further understanding the relationship between substance use and cognitive outcomes. Although urine toxicology screenings could have strengthened our findings, such sampling is considered intrusive by some and might have reduced the representability of our sample and increased attrition. Furthermore, these measures of sampling have limited validity and only for a narrow number of substances. We were aware of the possibility of underreporting, and therefore, assessments adopted a non-judgmental approach. Our impression was that details provided by patients was consistent with all other sources of information used in the project such as co-lateral information and patient files.

Longitudinal studies of FEP are useful in that they include baseline measures of neurocognitive performance thus minimizing the confounding effects of chronicity. Our study consists of a large representative cohort with patients followed up over a longer period than most other longitudinal FEP studies and with five repeated assessments of the cognitive domains.

The present study demonstrated differences in motor speed and verbal indices in patients who discontinued substance use early on in their course of treatment. This, as well as previous published results indicating that SUs reach levels as good as or better than NUs, conveys a powerful message to clinicians. Focusing on substance use early is crucial in order to maximize the likelihood of good outcomes.

## Ethics Statement

The study was carried out in accordance with the recommendations and has been approved by the Regional Committee for Research Ethics Health Region 2 (#S95189) and the Regional Committee for Research Ethics Health Region East (#1.2007.2177). Data inspectorate license: #96/3017-2, #2003/2052. The Regional Committee for science ethics region Sjælland: #1-01-83-0002-07. All subjects gave written informed consent in accordance with the Declaration of Helsinki. The protocol was approved by the Regional Commitees for Research ethics (see above).

## Author Contributions

SF, JJ, TL, IJ, PV, SO, IM, BR, ES, and UH took part in designing the study. TL, IJ, IM, SF, ES, UH, and WH collected the data. MW, WH, and BA performed the analyses. MW wrote the first draft of the manuscript with support from WH, JJ, as well as HS, KB, JR and JB. All authors critically reviewed the paper and approved the final version.

## Funding

This work was supported by Health West, Norway #911369; the Norwegian National Research Council #133897/320 and #154642/320, the Norwegian Department of Health and Social Affairs, and the National Council for Mental Health, Health and Rehabilitation #1997/41 and #2002/306, Rogaland County and Oslo County. This work was also funded by the Theodore and Vada Stanley Foundation and the Regional Health Research Foundation for Eastern Region, Denmark; Roskilde County, Helsefonden, Lundbeck Pharma, Eli Lily, and Janssen Cilag Pharmaceuticals Denmark; the National Alliance for Research on Schizophrenia and Depression (NARSAD) distinguished investigator award and NIM grant MH-01654 and a NARSAD young investigator award; and Health South East #2008001 and Health West #200202797-65 and #911313.

## Conflict of Interest Statement

SO has given advice to Otsuka and Lundbeck and lectured in meetings organized by those companies. SF has received an honorarium as a data consultant for RAND Corporation for a project sponsored by Janssen-Cilag pharmaceutical company. The remaining authors declare that the research was conducted in the absence of any commercial or financial relationships that could be construed as a potential conflict of interest.

## References

[1] Green MF What are the functional consequences of neurocognitive deficits in schizophrenia? Am J Psychiatry (1996) 153(3):321–30. 10.1176/ajp.153.3.321 8610818

[2] Kahn RS Keefe RS Schizophrenia is a cognitive illness: time for a change in focus. JAMA Psychiatry (2013) 70(10):1107–12. 10.1001/jamapsychiatry.2013.155 23925787

[3] Bahorik AL Newhill CE Eack SM Neurocognitive functioning of individuals with schizophrenia: using and not using drugs. Schizophr Bull (2014) 40(4):856–67. 10.1093/schbul/sbt099 PMC4059433 23884348

[4] Bora E Lin A Wood SJ Yung AR McGorry PD Pantelis C Cognitive deficits in youth with familial and clinical high risk to psychosis: a systematic review and meta-analysis. Acta Psychiatr Scand (2014) 130(1):1–15. 10.1111/acps.12261 24611632

[5] Morrens M Hulstijn W Sabbe B Psychomotor slowing in schizophrenia. Schizophr Bull (2007) 33(4):1038–53. 10.1093/schbul/sbl051 PMC2632327 17093141

[6] Bervoets C Docx L Sabbe B Vermeylen S Van Den Bossche MJ Morsel A The nature of the relationship of psychomotor slowing with negative symptomatology in schizophrenia. Cogn Neuropsychiatry (2014) 19(1):36–46. 10.1080/13546805.2013.779578 23725330

[7] Rajji TK Miranda D Mulsant BH Cognition, function, and disability in patients with schizophrenia: a review of longitudinal studies. Can J Psychiatry Rev (2014) 59(1):13–7. 10.1177/070674371405900104 PMC4079219 24444319

[8] Schaefer J Giangrande E Weinberger DR Dickinson D The global cognitive impairment in schizophrenia: consistent over decades and around the world. Schizophr Res (2013) 150(1):42–50. 10.1016/j.schres.2013.07.009 23911259 PMC4196267

[9] Reichenberg A Caspi A Harrington H Houts R Keefe RS Murray RM Static and dynamic cognitive deficits in childhood preceding adult schizophrenia: a 30-year study. Am J Psychiatry (2010) 167(2):160–9. 10.1176/appi.ajp.2009.09040574 PMC3552325 20048021

[10] Holmen A Juuhl-Langseth M Thormodsen R Melle I Rund BR Neuropsychological profile in early-onset schizophrenia-spectrum disorders: measured with the MATRICS battery. Schizophr Bull (2010) 36(4):852–9. 10.1093/schbul/sbn174 PMC2894602 19223656

[11] Mesholam-Gately RI Giuliano AJ Goff KP Faraone SV Seidman LJ Neurocognition in first-episode schizophrenia: a meta-analytic review. Neuropsychology (2009) 23(3):315–36. 10.1037/a0014708 19413446

[12] Carey KB Carey MP Simons JS Correlates of substance use disorder among psychiatric outpatients: focus on cognition, social role functioning, and psychiatric status. J Nervous Ment Dis (2003) 191(5):300–8. 10.1097/01.NMD.0000066152.87832.A9 PMC2430931 12819549

[13] McCleery A Addington J Addington D Substance misuse and cognitive functioning in early psychosis: a 2 year follow-up. Schizophr Res (2006) 88(1–3):187–91. 10.1016/j.schres.2006.06.040 16942863

[14] Sevy S Robinson DG Holloway S Alvir JM Woerner MG Bilder R Corerlates of substance abuse in patients with first-episode schizophrenia and schizoaffective disorder. Acta Psychiatr Scand (2001) 104:367–374. 10.1034/j.1600-0447.2001.00452.x 11722318

[15] Husa AP Moilanen J Murray GK Marttila R Haapea M Rannikko I Lifetime antipsychotic medication and cognitive performance in schizophrenia at age 43 years in a general population birth cohort. Psychiatry Res (2017) 247:130–8. 10.1016/j.psychres.2016.10.085 PMC5241225 27888683

[16] Hori H Noguchi H Hashimoto R Nakabayashi T Omori M Takahashi S Antipsychotic medication and cognitive function in schizophrenia. Schizophr Res (2006) 86(1–3):138–46. 10.1016/j.schres.2006.05.004 16793238

[17] Woodward ND Purdon SE Meltzer HY Zald DH A meta-analysis of neuropsychological change to clozapine, olanzapine, quetiapine, and risperidone in schizophrenia. Int J Neuropsychopharmacol (2005) 8(3):457–72. 10.1017/S146114570500516X 15784157

[18] Keefe RS Sweeney JA Gu H Hamer RM Perkins DO McEvoy JP Effects of olanzapine, quetiapine, and risperidone on neurocognitive function in early psychosis: a randomized, double-blind 52-week comparison. Am J Psychiatry (2007) 164(7):1061–71. 10.1176/ajp.2007.164.7.1061 17606658

[19] Crespo-Facorro B Rodriguez-Sanchez JM Perez-Iglesias R Mata I Ayesa R Ramirez-Bonilla M Neurocognitive effectiveness of haloperidol, risperidone, and olanzapine in first-episode psychosis: a randomized, controlled 1-year follow-up comparison. J Clin Psychiatry (2009) 70(5):717–29. 10.4088/JCP.08m04634 19389335

[20] Barnett JH Werners U Secher SM Hill KE Brazil R Masson K Substance use in a population-based clinic sample of people with first-episode psychosis. Br J Psychiatry (2007) 190:515–20. 10.1192/bjp.bp.106.024448 17541112

[21] Wade D Harrigan S Edwards J Burgess PM Whelan G McGorry PD Substance misuse in first-episode psychosis: 15-month prospective follow-up study. Br J Psychiatry (2006) 189:229–34. 10.1192/bjp.bp.105.017236 16946357

[22] Wisdom JP Manuel JI Drake RE Substance use disorder among people with first-episode psychosis: a systematic review of course and treatment. Psychiatr Serv (2011) 62(9):1007–12. 10.1176/ps.62.9.pss6209_1007 PMC3575521 21885577

[23] D’Souza C Cho H Perry E A cannabinoid model for psychosis, dopamine-cannabinoid interaction and implications for schizophrenia. Cambridge: Cambridge University Press (2004).

[24] D'Souza DC Abi-Saab WM Madonick S Forselius-Bielen K Doersch A Braley G Delta-9-tetrahydrocannabinol effects in schizophrenia: implications for cognition, psychosis, and addiction. Biol Psychiatry (2005) 57(6):594–608. 10.1016/j.biopsych.2004.12.006 15780846

[25] Rabin RA Zakzanis KK George TP The effects of cannabis use on neurocognition in schizophrenia: a meta-analysis. Schizophr Res (2011) 128(1–3):111–6. 10.1016/j.schres.2011.02.017 21420282

[26] Thoma P Daum I Comorbid substance use disorder in schizophrenia: a selective overview of neurobiological and cognitive underpinnings. Psychiatry Clin Neurosci (2013) 67(6):367–83. 10.1111/pcn.12072 23890122

[27] Loberg EM Helle S Nygard M Berle JO Kroken RA Johnsen E The Cannabis Pathway to non-affective psychosis may reflect less neurobiological vulnerability. Front Psychiatry (2014) 5:159. 10.3389/fpsyt.2014.00159 25477825 PMC4235385

[28] Wobrock T Falkai P Schneider-Axmann T Hasan A Galderisi S Davidson M Comorbid substance abuse in first-episode schizophrenia: effects on cognition and psychopathology in the EUFEST study. Schizophr Res (2013) 147(1):132–9. 10.1016/j.schres.2013.03.001 23537477

[29] Yücel M Bora E Lubman DI Solowij N Brewer WJ Cotton SM The impact of cannabis use on cognitive functioning in patients with schizophrenia: a meta-analysis of existing findings and new data in a first-episode sample. Schizophr Bull (2012) 38(2):316–30. 10.1093/schbul/sbq079 PMC3283159 20660494

[30] Coulston CM Perdices M Tennant CC The neuropsychology of cannabis and other substance use in schizophrenia: review of the literature and critical evaluation of methodological issues. Aust N Z J Psychiatry (2007) 41(11):869–84. 10.1080/00048670701634952 17924240

[31] Rodriguez-Jimenez R Bagney A Martinez-Gras I Ponce G Sanchez-Morla EM Aragües M Executive function in schizophrenia: influence of substance use disorder history. Schizophr Res (2010) 118(1–3):34–40. 10.1016/j.schres.2009.09.025 19854622

[32] Leeson VC Harrison I Ron MA Barnes TRE Joyce EM The Effect of cannabis use and cognitive reserve on age at onset and psychosis outcomes in first-episode schizophrenia. Schizophr Bull (2012) 38(4):873–80. 10.1093/schbul/sbq153 PMC3406524 21389110

[33] Albus M Hubmann W Mohr F Hecht S Hinterberger-Weber P Seitz NN Neurocognitive functioning in patients with first-episode schizophrenia: results of a prospective 5-year follow-up study. Eur Arch Psychiatry Clin Neurosci (2006) 256(7):442–51. 10.1007/s00406-006-0667-1 17031490

[34] Stirling J White C Lewis S Hopkins R Tantam D Huddy A Neurocognitive function and outcome in first-episode schizophrenia: a 10-year follow-up of an epidemiological cohort. Schizophr Res (2003) 65(2–3):75–86. 10.1016/S0920-9964(03)00014-8 14630300

[35] Leeson VC Robbins TW Matheson E Hutton SB Ron MA Barnes TR Discrimination learning, reversal, and set-shifting in first-episode schizophrenia: stability over six years and specific associations with medication type and disorganization syndrome. Biol Psychiatry (2009) 66(6):586–93. 10.1016/j.biopsych.2009.05.016 PMC2734076 19576575

[36] Hoff AL Svetina C Shields G Stewart J DeLisi LE Ten year longitudinal study of neuropsychological functioning subsequent to a first episode of schizophrenia. Schizophr Res (2005) 78(1):27–34. 10.1016/j.schres.2005.05.010 15964177

[37] Gold S Arndt S Nopoulos P O’Leary DS Andreasen NC Longitudinal study of cognitive function in first-episode and recent-onset schizophrenia. Am J Psychiatry (1999) 156(9):1342–8. 10.1176/ajp.156.9.1342 10484943

[38] Oie M Sundet K Rund BR Neurocognitive decline in early-onset schizophrenia compared with ADHD and normal controls: evidence from a 13-year follow-up study. Schizophr Bull (2010) 36(3):557–65. 10.1093/schbul/sbn127 PMC2879697 18801881

[39] Barder HE Sundet K Rund BR Evensen J Haahr U Velden Hegelstad W Ten year neurocognitive trajectories in first-episode psychosis. Front Hum Neurosci (2013) 7:643. 10.3389/fnhum.2013.00643 24109449 PMC3791439

[40] Szoke A Trandafir A Dupont ME Meary A Schurhoff F Leboyer M Longitudinal studies of cognition in schizophrenia: meta-analysis. Br J Psychiatry (2008) 192(4):248–57. 10.1192/bjp.bp.106.029009 18378982

[41] Bozikas VP Andreou C Longitudinal studies of cognition in first episode psychosis: a systematic review of the literature. Aust N Z J Psychiatry (2011) 45(2):93–108. 10.3109/00048674.2010.541418 21320033

[42] Rund BR A review of longitudinal studies of cognitive functions in schizophrenia patients. Schizophr Bull (1998) 24(3):425–35. 10.1093/oxfordjournals.schbul.a033337 9718634

[43] Addington J Addington D Patterns, prdictors and impact of substance use in early psychosis: a longitudinal study. Acta Psychiatr Scand (2007) 115:304–9. 10.1111/j.1600-0447.2006.00900.x 17355521

[44] Drake RE McHugo GJ Xie HY Fox M Packard J Helmstetter B Ten-year recovery outcomes for clients with co-occurring schizophrenia and substance use disorders. Schizophr Bull (2006) 32(3):464–73. 10.1093/schbul/sbj064 PMC2632251 16525088

[45] Mullin K Gupta P Compton MT Nielssen O Harris A Large M Does giving up substance use work for patients with psychosis? A systematic meta-analysis. Aust N Z J Psychiatry (2012) 46(9):826–39. 10.1177/0004867412440192 22368242

[46] González-Pinto A Alberich S Barbeito S Gutierrez M Vega P Ibáñez B Cannabis and first-episode psychosis: different long-term outcomes depending on continued or discontinued use. Schizophr Bull (2011) 37(3):631–9. 10.1093/schbul/sbp126 PMC3080669 19915168

[47] Weibell MA Hegelstad WTV Auestad B Bramness J Evensen J Haahr U The effect of substance use on 10-year outcome in first-episode psychosis. Schizophr Bull (2017) 11:843–51. 10.1093/schbul/sbw179 PMC5472130 28199703

[48] Larsen TK Melle I Auestad B Friis S Haahr U Johannessen JO Substance abuse in first-episode non-affective psychosis. Schizophr Res (2006) 88(1–3):55–62. 10.1016/j.schres.2006.07.018 16971092

[49] Rund BR Barder HE Evensen J Haahr U Velden Hegelstad W Joa I Neurocognition and duration of psychosis: a 10-year follow-up of first-episode patients. Schizophr Bull (2016) 42(1):87–95. 10.1093/schbul/sbv083 26101305 PMC4681546

[50] Rund BR Melle I Friis S Johannessen JO Larsen TK Midbøe LJ The course of neurocognitive functioning in first-episode psychosis and its relation to premorbid adjustment, duration of untreated psychosis, and relapse. Schizophr Res (2007) 91(1–3):132–40. 10.1016/j.schres.2006.11.030 17258891

[51] Melle I Larsen TK Haahr U Friis S Johannessen JO Opjordsmoen S Reducing the duration of untreated firs-episode psychosis: effects on clinical presentation. Arch Gen Psychiatry (2004) 61:143–50. 10.1001/archpsyc.61.2.143 14757590

[52] American Psychiatric Association Diagnostic and statistical manual of mental disorders. 4th ed Washington DC: American Psychiatric Association (2000).

[53] Kay SR Fiszbein A Opler LA The positive and negative syndrome scale (PANSS) for schizophrenia. Schizophr Bull (1987) 13(2):261–76. 10.1093/schbul/13.2.261 3616518

[54] Pedersen G Hagtvet K Karterud S Generalizability studies of the Global Asssessment of Functionin- Split version. Compr Psychiatry (2007) 48(1):88–94. 10.1016/j.comppsych.2006.03.008 17145287

[55] Bentsen H Notland TH Boye B Bjørge H Lersbryggen AB Oskarsson KH The interrater reliability of the Positive and Negative Syndrome Scale (PANSS). Int J Methods Psychiatr Res (1996) 6:227–35. 10.1002/(SICI)1234-988X(199612)6:4<227::AID-MPR162>3.3.CO;2-A

[56] Cannon-Spoor HE Potkin SG Wyatt RJ Measurement of premorbid adjustment in chronic schizophrenia. Schizophr Bull (1982) 8(3):470–84. 10.1093/schbul/8.3.470 7134891

[57] Larsen TK Friis S Haahr U Johannessen JO Melle I Opjordsmoen S Premorbid adjustment in first-episode non-affective psychosis: distinct patterns of pre-onset course. Br J Psychiatry (2004) 185:108–15. 10.1192/bjp.185.2.108 15286061

[58] Delis DC Kramer JH Kapla E Ober BA The California Verbal Learning Test. 2nd ed San Antonio Texas: The Psychological Corporation (2000).

[59] Heaton RK Chelune GJ Talley JL Kay GG Curtiss G Wisconsin Card Sorting Test Manual: Revised and Expanded. New York NY: Oxford University Press (1993).

[60] Strauss E Sherman EMS Spreen O A Compendium of Neuropsychological Tests: Administration, Norms and Commentary, 2nd ed New York: Oxford University Press (1998).

[61] Friis S Sundet K Rund BR Vaglum P McGlashan TH Neurocognitive dimensions characterising patients with first-episode psychosis. Br J Psychiatry Suppl (2002) 181:s85–s90. 10.1016/S0924-9338(02)80241-3 12271806

[62] Friis S Larsen TK Melle I Opjordsmoen S Johannessen JO Haahr U Methodological pitfalls in early detection studies - the NAPE Lecture 2002. Acta Psychiatr Scand (2003) 107(1):3–9. 10.1034/j.1600-0447.2003.02600.x 12558535

[63] Hegelstad WT Larsen TK Auestad B Evensen J Haahr U Joa I Long-term follow-up of the TIPS early detection in psychosis study: effects on 10-year outcome. Am J Psychiatry (2012) 169(4):374–80. 10.1176/appi.ajp.2011.11030459 22407080

[64] Mueser KT Noordsy DL Drake RE Fox L Integrated treatment for dual disorders. New York: The Guilford Press (2003).

[65] Baeza I Graell M Moreno D Castro-Fornieles J Parellada M González-Pinto A Cannabis use in children and adolescents with first episode psychosis: influence on psychopathology and short-term outcome (CAFEPS study). Schizophr Res (2009) 113(2–3):129–37. 10.1016/j.schres.2009.04.005 19427172

[66] Lambert M Conus P Lubman DI Wade D Yuen H Moritz S The impact of substance use disorders on clinical outcome in 643 patients with first-episode psychosis. Acta Psychiatr Scand (2005) 112(2):141–8. 10.1111/j.1600-0447.2005.00554.x 15992396

[67] Grech A Van Os J Jones PB Lewis SW Murray RM Cannabis use and outcome of recent onset psychosis. Eur Psychiatry (2005) 20(4):349–53. 10.1016/j.eurpsy.2004.09.013 16018929

[68] Archie S Rush BR Akhtar-Danesh N Norman R Malla A Roy P Substance use and abuse in first-episode psychosis: prevalence before and after early intervention. Schizophr Bull (2007) 33(6):1354–63. 10.1093/schbul/sbm011 PMC2779870 17337748

[69] SPSS for Windows (11.5.1) [computer program]. Version. Chicago: SPSS inc; 2001.

[70] R: A language and environment for statistical computing [computer program]. Version. Vienna, Austria; 2017.

[71] Strakowski S DelBello M Fleck D Adler C Anthenelli R Arnold L Effects of co-occurring cannabis use disorders on the course of bipolar disorder after a first hspitalization for mania. Arch Gen Psychiatry (2007) 64(1):57–64. 10.1001/archpsyc.64.1.57 17199055

[72] Hoff AL Svetina C Maurizio AM Crow TJ Spokes K DeLisi LE Familial cognitive deficits in schizophrenia. Am J Med Genet B Neuropsychiatr Genet (2005) 133B(1):43–9. 10.1002/ajmg.b.30120 15635688

[73] Chen YL Chen YH Lieh-Mak F Semantic verbal fluency deficit as a familial trait marker in schizophrenia. Psychiatry Res (2000) 95(2):133–48. 10.1016/S0165-1781(00)00166-9 10963799

