# Supplementary material for: Early Substance Use Cessation Improves Cognition—10 Years Outcome in First-Episode Psychosis Patients
Source: Front Psychiatry. 2019 Jul 12;10:495. doi: 10.3389/fpsyt.2019.00495 (PMC6640174; doi:10.3389/fpsyt.2019.00495)
Supplement: Supplementary file 1 [file DataSheet_1.pdf]

Supplementary material

Mean values and 95% CI for the neurocognitive indices shown over time in non-users (0, black), stop-users (1, red), persistent users (2, green) and episodic users (3, blue).

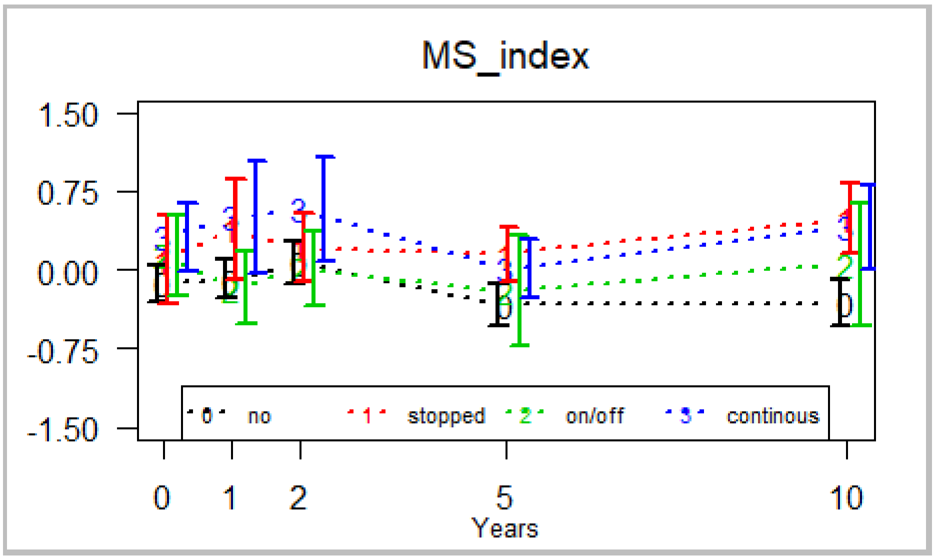

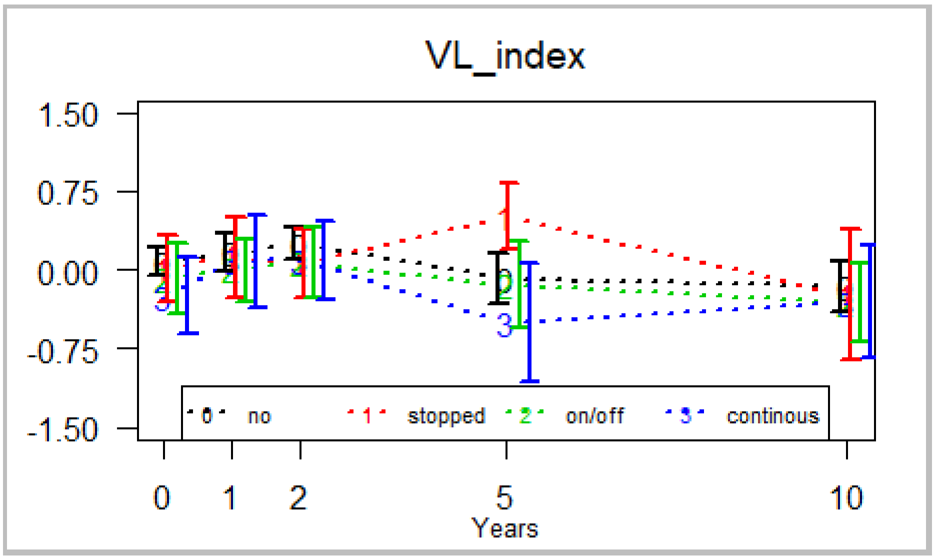

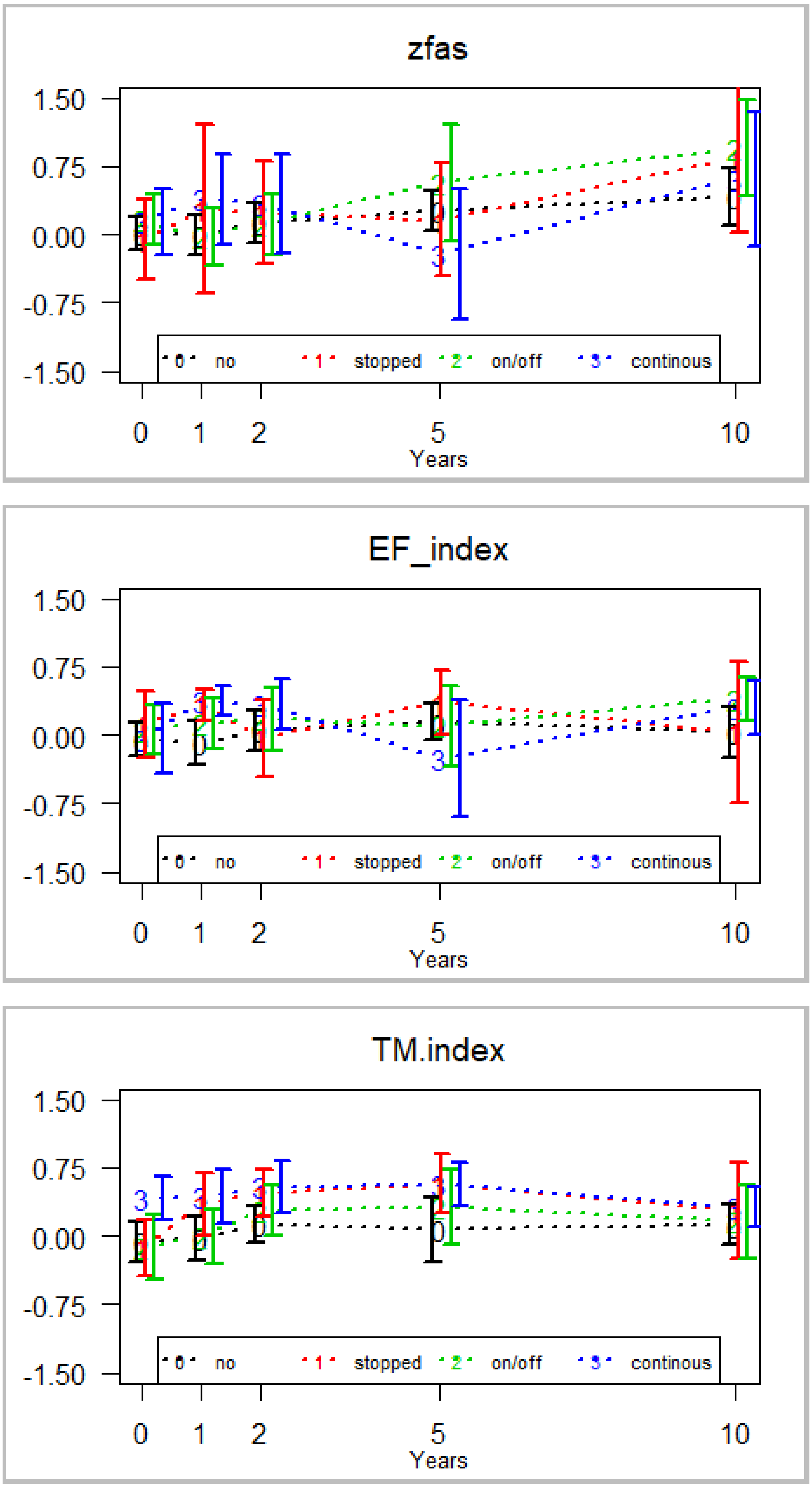
zfas
1.50
0.75
0.00
-0.75
-1.50
0 no
1 stopped
2 on/off
3 continous
0
1
2
5
10
Years
EF_index
1.50
0.75
0.00
-0.75
-1.50
0 no
1 stopped
2 on/off
3 continous
0
1
2
5
10
Years
TM.index
1.50
0.75
0.00
-0.75
-1.50
0 no
1 stopped
2 on/off
3 continous
0
1
2
5
10
Years

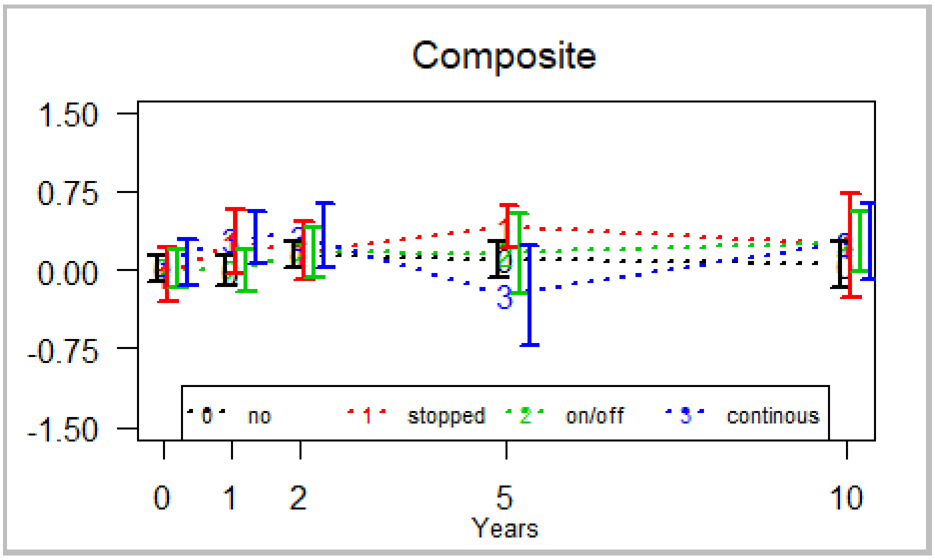

Composite
1.50
0.75
0.00
-0.75
-1.50
0
1
2
5
10
Years
0 no
1 stopped
2 on/off
3 continous
